# Participant Engagement in Microrandomized Trials of mHealth Interventions: Scoping Review

**DOI:** 10.2196/44685

**Published:** 2023-05-22

**Authors:** Utek Leong, Bibhas Chakraborty

**Affiliations:** 1 Department of Psychology National University of Singapore Singapore Singapore; 2 Centre for Quantitative Medicine and Program in Health Services and Systems Research Duke-NUS Medical School National University of Singapore Singapore Singapore; 3 Department of Statistics and Data Science National University of Singapore Singapore Singapore; 4 Department of Biostatistics and Bioinformatics Duke University Durham, NC United States

**Keywords:** microrandomized trials, engagement, adherence, mobile health, mHealth interventions, mobile phone

## Abstract

**Background:**

Microrandomized trials (MRTs) have emerged as the gold standard for the development and evaluation of multicomponent, adaptive mobile health (mHealth) interventions. However, not much is known about the state of participant engagement measurement in MRTs of mHealth interventions.

**Objective:**

In this scoping review, we aimed to quantify the proportion of existing or planned MRTs of mHealth interventions to date that have assessed (or have planned to assess) engagement. In addition, for the trials that have explicitly assessed (or have planned to assess) engagement, we aimed to investigate how engagement has been operationalized and to identify the factors that have been studied as determinants of engagement in MRTs of mHealth interventions.

**Methods:**

We conducted a broad search for MRTs of mHealth interventions in 5 databases and manually searched preprint servers and trial registries. Study characteristics of each included evidence source were extracted. We coded and categorized these data to identify how engagement has been operationalized and which determinants, moderators, and covariates have been assessed in existing MRTs.

**Results:**

Our database and manual search yielded 22 eligible evidence sources. Most of these studies (14/22, 64%) were designed to evaluate the effects of intervention components. The median sample size of the included MRTs was 110.5. At least 1 explicit measure of engagement was included in 91% (20/22) of the included MRTs. We found that objective measures such as system usage data (16/20, 80%) and sensor data (7/20, 35%) are the most common methods of measuring engagement. All studies included at least 1 measure of the physical facet of engagement, but the affective and cognitive facets of engagement have largely been neglected (only measured by 1 study each). Most studies measured engagement with the mHealth intervention (Little e) and not with the health behavior of interest (Big E). Only 6 (30%) of the 20 studies that measured engagement assessed the determinants of engagement in MRTs of mHealth interventions; notification-related variables were the most common determinants of engagement assessed (4/6, 67% studies). Of the 6 studies, 3 (50%) examined the moderators of participant engagement—2 studies investigated time-related moderators exclusively, and 1 study planned to investigate a comprehensive set of physiological and psychosocial moderators in addition to time-related moderators.

**Conclusions:**

Although the measurement of participant engagement in MRTs of mHealth interventions is prevalent, there is a need for future trials to diversify the measurement of engagement. There is also a need for researchers to address the lack of attention to how engagement is determined and moderated. We hope that by mapping the state of engagement measurement in existing MRTs of mHealth interventions, this review will encourage researchers to pay more attention to these issues when planning for engagement measurement in future trials.

## Introduction

### Background

In the past decade, digital solutions that leverage mobile technologies to improve health and well-being have become increasingly popular and have emerged as promising adjuncts to traditional health care provision [1]. These so-called mobile health (mHealth) interventions generally involve the use of mobile technologies such as mobile apps, SMS text messaging, and wearable devices to improve patient health outcomes by delivering health-related intervention content. Mounting evidence suggests that mHealth interventions are largely effective for treating chronic health conditions [2,3] and for preventing unhealthy behaviors [4]. Effectiveness aside, it is not difficult to see why mHealth interventions are so popular; mHealth interventions are highly scalable and cost-efficient [1]. High rates of mobile ownership worldwide also signal the potential for mHealth interventions to reach a diverse audience, including the underserved; however, we must acknowledge that there are barriers to access (such as the lack of internet access) that prevent mHealth interventions from being truly equitable [5].

Recently, more sophisticated mHealth interventions have been proposed to take advantage of the technological advances in mobile technology. These novel interventions (such as just-in-time adaptive interventions) tend to be multicomponent, that is, they tend to involve the manipulation of ≥2 components hypothesized to have a treatment effect. They also tend to be adaptive, in the sense that components of the intervention (eg, its content and timing of delivery) can change in response to some input data provided by the user (tailoring data collected from surveys or sensors). To make this concrete, let us consider a hypothetical mHealth intervention designed to reduce the severity of depression symptoms by sending daily motivational messages via SMS text messaging. The intervention is said to be multicomponent if both message content and timing of SMS delivery are thought to be *active* ingredients that can influence depression symptom severity. Such an intervention could be made adaptive if daily message content is tailored to the participant’s mood the night before such that if a given participant had high negative mood the night before, a more strongly worded motivational message would be sent the next day. Unfortunately, conventional randomized controlled trials (RCTs) cannot be used to develop and optimize these interventions because they do not allow researchers to separate the treatment effect of individual treatment components from the overall treatment effect. In addition, RCTs do not allow researchers to investigate time-varying effects, which is of interest when the goal is to identify the optimal time to administer an intervention component [6]. Therefore, if the RCT design is used to study the aforementioned hypothetical mHealth intervention, we will only be able to estimate the *overall* treatment effect of sending motivational messages on depression symptom severity and not the specific treatment effect of message content and timing of SMS delivery on the severity of depressive symptoms.

To address these limitations of the RCT design, several cutting-edge trial designs have been proposed in recent years. The microrandomized trial (MRT) design in particular has gained considerable traction as a way to optimize multicomponent and adaptive mHealth interventions (including but not limited to just-in-time adaptive interventions) [6-9]. Essentially, the MRT design involves the repeated random assignment of participants to different intervention options of a single or multiple intervention components; therefore, an MRT of our hypothetical multicomponent motivational SMS text messaging intervention would entail repeatedly randomizing participants to receive *different types of motivational messages* at *different times daily*. This repeated random assignment then facilitates the estimation of the time-varying causal effects of each specific treatment component [6], that is, we can estimate the treatment effect of message content and timing of SMS text message delivery on the severity of depressive symptoms. Therefore, unlike RCTs, MRTs allow researchers to investigate the effectiveness of specific components of mHealth interventions, which could be informative for theory, future research, and intervention optimization. Notably, RCTs and MRTs are not mutually exclusive. One additional benefit of the MRT design is that it can be easily embedded within the treatment arm of a conventional RCT; therefore, the overall treatment effect and the effect of specific intervention components can be tested simultaneously.

Regardless of the trial design used, the measurement of participant engagement is integral to understanding the feasibility of mHealth interventions. This is because engagement with the constituent digital or nondigital intervention stimuli and tasks of an mHealth intervention is necessary for the individual to experience the intended distal health outcomes of the intervention [10,11]. The measurement of engagement, however, is not straightforward. Engagement, like many other psychological constructs, is an abstract and fuzzy concept that is not directly measurable (unlike, for example, the measurement of height). To measure engagement, researchers must first operationalize engagement, that is, define engagement in measurable terms [12]. To unpack how exactly engagement with mHealth interventions can be operationalized, it is instructive to consider *how* engagement can be measured, *which* kinds of engagement can be measured, and *what* levels of engagement can be measured.

### Measures of Engagement

According to Yardley et al [13] and then Short et al [14], there are 7 methods of engagement measurement that researchers can use to obtain a sense of participant engagement in their digital interventions: self-report questionnaires, ecological momentary assessments (EMAs), qualitative methods, system usage data, sensor data, social media data, and psychophysiological measures. The measurement of engagement via self-report questionnaires and EMAs involves directly asking participants to report (via single items or questionnaires) their subjective experience of using the digital intervention or their use of the intervention. Qualitative methods of engagement, by contrast, involve the inference of engagement from qualitative sources (such as written responses and semistructured interviews). Measuring engagement via system usage data involves the quantification of how the digital intervention is used through metrics including, but not limited to, the number of log-ins, time spent on the intervention, and number of modules viewed. Engagement can also be measured by analyzing passively collected social media and sensor data if social media and sensors (eg, pedometers and heart rate sensors) are a feature of the intervention. Finally, psychophysiological measures of engagement involve the use of measures such as electroencephalography, eye tracking, or functional magnetic resonance imaging to infer engagement from neural and physiological activity.

### Facets of Engagement

Engagement is thought to be a multifaceted construct composed of 3 distinct facets—physical, affective, and cognitive [11,14]. The physical facet of engagement refers to the “actual performance of an activity or task” [11]. The affective facet by contrast is thought to capture “a wide range of positive affective reactions to a task or activity, from feeling pride, enthusiasm, and satisfaction, to affective states that may underlie more enduring experiences of attachment, identification, and commitment” [11]. Finally, the cognitive facet of engagement is thought to refer to “selective attention and processing of information related to a task or activity” [11]. These facets represent distinct *kinds* of engagement that can be measured in mHealth interventions.

### Levels of Engagement

When discussing the measurement of engagement in digital interventions, it is crucial to ask the question, “engagement with what?” [11]. This is because engagement measures can either be measures of engagement with the features and the active ingredients of the intervention or engagement with the health behavior of interest. Formally, Cole-Lewis et al [15] termed engagement with the mHealth intervention as “Little e” and engagement with the health behavior of interest as “Big E”; elsewhere, the terms microengagement and macroengagement are used instead [13]. In essence, Little e and Big E represent 2 distinct levels of engagement, where the 7 methods of engagement outlined in the *Measures of Engagement* section can be applied to measure participant engagement in the mHealth intervention context.

### This Study

Given the importance of engagement to mHealth interventions, researchers have endeavored to understand how engagement has been conceptualized and operationalized in studies evaluating mHealth interventions. For instance, Pham et al [16] recently reviewed how engagement has been defined and measured in mHealth apps for chronic conditions. Perski et al [10], by contrast, reviewed how engagement was conceptualized in digital behavior change interventions (their review was not limited to mHealth interventions; it included other digital interventions). Other recent reviews evaluated the measurement of engagement in mHealth interventions designed for specific health conditions [17,18]. However, none of these reviews examined mHealth interventions evaluated by MRTs, perhaps owing to the relative infancy of the trial design. Thus, not much is known about the state of participant engagement measurement in MRTs of mHealth interventions. Furthermore, it is not yet known what kinds of factors have been studied as determinants of engagement in these MRTs.

Therefore, we conducted a scoping review to map this relatively new research area. We chose to conduct a scoping review as we expected that only a handful of mHealth intervention MRTs have been conducted to date—too few to be meaningfully synthesized with a systematic review. This scoping review aimed to address 3 review questions:

1. What proportion of existing (or planned) MRTs of mHealth interventions to date have assessed (or have planned to assess) engagement?
2. How has engagement been operationalized in existing (or planned) MRTs of mHealth interventions that have assessed (or have planned to assess) engagement?
3. In existing (or planned) MRTs of mHealth interventions that have assessed (or have planned to assess) engagement, what kind of factors have been studied as determinants of engagement?

## Methods

### Protocol and Registration

The protocol for this scoping review was developed using the Joanna Briggs Institute Manual for Evidence Synthesis [19] and was designed to ensure adherence to the PRISMA-ScR (Preferred Reporting Items for Systematic Reviews and Meta-Analysis extension for Scoping Reviews) guidelines [20]. The protocol and its appendices were prospectively registered with the Open Science Framework (OSF) on June 30, 2022 [21].

### Eligibility Criteria

We prioritized the inclusion of papers published in peer-reviewed journals. We included preprints, trial protocols, and dissertations (this was mistakenly left out of the “Types of Sources” section of our protocol [21]) only if no corresponding peer-reviewed journal articles were available. Conference abstracts were excluded from this scoping review.

All papers fulfilling these criteria to date were considered for inclusion if they were written in English and if they reported MRTs of mHealth interventions. We also included any secondary analyses of mHealth intervention engagement data collected from an MRT if the primary analysis (if available) did not report the assessment of engagement in detail. We defined mHealth interventions as any intervention designed to improve health outcomes through (though not limited to) the modification of health behavior (such as physical activity or treatment adherence), the improvement of patient knowledge, health monitoring, and the reduction of psychological distress via mobile technology such as SMS text messaging; mobile phone apps; or devices (including but not limited to smartwatches, wearables, and sensors) [1].

As the review’s objectives concerned the assessment of engagement in MRTs of mHealth interventions, we included all studies in which authors *explicitly* attempted or claimed to quantitatively or qualitatively measure the participation in or use of mHealth interventions directly (by measuring participation in or performance of mHealth intervention activities or components) or indirectly (using measurements derived from non–intervention-related activities or components as a proxy), regardless of how they actually defined and measured engagement (eg, if they use alternative terms like adherence).

### Information Sources and Search Strategy

We conducted a broad search for all published MRTs of mHealth interventions to date (the search was initially conducted on July 13, 2022, and again on September 28, 2022) by searching the following 5 bibliographic databases: MEDLINE (via PubMed), Embase, PsycINFO, CINAHL, and Cochrane Library. The search strategy was originally developed for MEDLINE, and we consulted an academic librarian from the National University of Singapore to ensure that the search strategy was comprehensive and sound. This search strategy was then translated for the 4 other databases (only syntax was changed to accommodate differences in search engines; keywords remained the same). Although only 1 broad search was eventually performed, it must be noted that we registered 2 separate searches in our protocol—1 for all published MRTs of mHealth interventions to date and 1 fine-grained search for MRTs of mHealth interventions that have assessed (or have planned to assess) engagement. During our search process, we realized that the latter search was redundant as it was nested within the former (because we used the Boolean operator AND between the mHealth intervention search terms and the engagement-related search terms). Therefore, we condensed the 2 planned searches into 1 by using the Boolean operator OR instead, such that our database searches indexed any MRTs that mentioned mHealth interventions or engagement-related terms. The comprehensive search strategies for all 5 databases (and their respective previous iterations) can be found on OSF [21].

To search for gray literature and unpublished studies, we searched the reference lists of included studies for any additional sources not indexed by our database search. We also posted an open call for unpublished MRTs of mHealth interventions on Twitter and contacted known experts of the MRT design to request unpublished and file-drawered studies. Finally, we performed a search (similarly, this search was initially conducted on July 13, 2022, and again on September 28, 2022) of MRTs of mHealth intervention on 2 preprint servers (PsyArXiv and medRxiv; we added this search during our search process to ensure the comprehensiveness of our gray literature search) and on 2 clinical trial registries, ClinicalTrials.gov (as detailed in our protocol) and the International Clinical Trials Registry Platform (this was added during the search process as well). The following search terms were used: “microrandomised,” “microrandomized,” “micro-randomised,” and “micro-​randomized.”

### Selection of Sources of Evidence

The results of the searches described in the previous section were imported into EndNote (version 20; Clarivate; we did not use Zotero as planned because of technical difficulties) for source selection and screening. The titles and abstracts of all potential evidence sources were first screened for eligibility. Eligible sources were then subjected to a full-text screening. Before the 2 screening stages, both authors discussed a subset of the search results (5 titles and abstracts and 4 full-text articles) to calibrate the selection of evidence sources. UL performed the screening using the eligibility criteria, and BC verified the screening at both stages. Any disagreements were resolved by consensus.

### Data Charting Process and Data Items

As described in our protocol [21], we developed an initial data extraction form (a Microsoft Excel [Microsoft Corporation] spreadsheet) to chart the data from eligible evidence sources to obtain the information necessary to answer our review questions. Both authors (UL and BC) piloted this initial data extraction form with 4 included articles to calibrate the charting process and to ensure that relevant data items were captured by the form. This form was continuously updated during the charting process through the discussion of the extracted results. UL performed data charting, and BC verified the charted data for all eligible evidence sources. Any disagreements were resolved by consensus.

The initial data collection form was designed to abstract the following information from each paper: whether the paper described a primary or secondary analysis of MRT data, type of paper, sample size of the MRT, sample characteristics, purpose of the study, type of mHealth intervention assessed, mode of delivery for the mHealth intervention, if engagement was or will be assessed, how engagement was operationalized (if assessed), if determinants of engagement were or will be assessed, and (if any) what determinants of engagement were or will be assessed; for comprehensiveness, we also charted any moderating variables and control variables (covariates) assessed.

After piloting the form and during the charting process, we included additional data items to capture the following information: primary and secondary (if any) outcomes of the study, randomization design of the MRT, frequency of microrandomization, and the overall duration of the MRT. The final version of the data extraction form is available on OSF [21].

### Synthesis of Results

To quantify the proportion of existing and planned MRTs of mHealth interventions to date that have assessed (or have planned to assess) engagement, we tabulated the number of evidence sources charted to have assessed or planned to assess engagement. The included evidence sources were grouped by their purpose and presented in a tabular format. The mHealth interventions of each included evidence source were categorized based on their target. We used the following categories: mental health promotion, smoking cessation, physical activity promotion, sleep improvement, dietary lapse prevention or weight management behavior promotion, gambling reduction, and alcohol use reduction.

To understand how engagement has been operationalized in MRTs of mHealth interventions, we sought to determine *how* included evidence sources measured engagement, *which* kinds of engagement they measured, and *what* levels of engagement they measured*.* To determine *how* engagement has been measured, we classified *explicit* measures of engagement from each included source according to the methods of engagement measurement outlined by Short et al [14] described in the *Introduction* section. We combined the self-report questionnaires and EMA categories for parsimony, as they are largely similar methods of measuring engagement. To determine *which* kinds of engagement have been measured, we classified *explicit* measures of engagement by the facets (physical, affective, or cognitive) of engagement they appear to measure [11]. Finally, to determine *what* levels of engagement have been measured, we classified the *explicit* measures of engagement from each included source as Little e or Big E measures [15].

To identify the factors that have been studied as determinants of engagement in MRTs of mHealth interventions, we extracted the variables of interest, moderators, and covariates from each model (with a measure of engagement as the dependent variable) tested in each included source. We then organized these variables into the following categories: notification related (eg, type of prompt sent), time related (eg, days since the start of the intervention or day of the week), psychological, societal, health behavior related (eg, alcohol use), contextual (eg, location data), physiological (heart rate), demographic, anthropometric (eg, weight change), or task related (eg, intervention-related activities).

## Results

### Selection of Sources of Evidence

**Figure 1:**
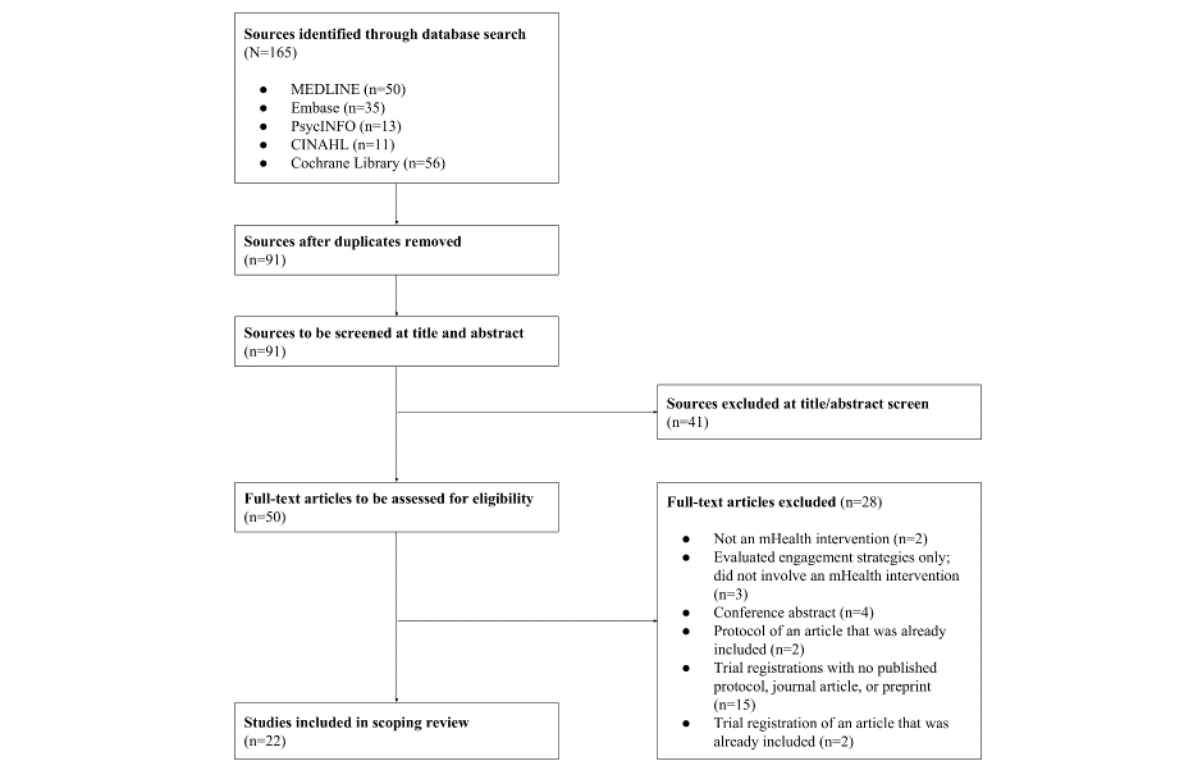
Evidence source selection flow diagram.

A total of 165 evidence sources were retrieved by our database search. After removing duplicates, 91 evidence sources were retained for further screening. During the title and abstract screening, 41 sources were excluded. Of the remaining 50 evidence sources, 28 were excluded at the full-text screening (Figure 1).

Notably, 17 of these sources excluded at full-text screening were trial registrations (a total of 19 trial registrations were retrieved by our database search of the Cochrane Library). A total of 15 (88%) of these 17 sources had no published protocol, journal article, or preprint; we performed a manual Google search of their respective trial identification numbers to confirm this. In total, 2 (12%) of these 17 sources were duplicate trial registrations, that is, a corresponding protocol, journal, article, or preprint for each registration was already indexed by our database search. Therefore, only 22 evidence sources identified by our database search were considered eligible for this scoping review. No additional studies were identified and included from our planned searches of gray literature and unpublished studies.

### Characteristics of Sources of Evidence

All charted data described in the preceding section are available on OSF [21] and Multimedia Appendix 1 [22-43]. We present a subset of the charted data that are pertinent to our review questions.

Table 1 details the characteristics of each included evidence source. Of the 22 included sources, 12 (54%) were published journal articles, 8 (36%) were trial protocols, 1 (5%) was a preprint, and 1 (5%) was a dissertation. Only 1 evidence source was a secondary analysis of MRT data [22]. All included sources were published between 2018 and 2022. More than half of the included sources (14/22, 64%) were designed to evaluate the effect of intervention components. Physical activity promotion was the most common target of the mHealth interventions (8/22, 36%). Interventions were largely delivered via smartphone apps. The median sample size of the included MRTs was 110.5.

**Table 1:** Characteristics of included evidence sources.

Source and intervention type				Mode of delivery		Engagement assessed?
**Evaluate effect of intervention components**						
	Aguilera et al [23], 2021	Mental health promotion	SMS		Yes	
	Battalio et al [24], 2021	Smoking cessation	App		Yes	
	Figueroa et al [25], 2022	Physical activity promotion	SMS, app		No	
	Goldstein et al [26], 2021	Dietary lapse prevention or weight management behavior promotion	App		Yes	
	Klasnja et al [27], 2021	Physical activity promotion	SMS		Yes	
	Klasnja et al [28], 2019	Physical activity promotion	App		Yes	
	Kramer et al [29], 2020	Physical activity promotion	App		Yes	
	Latham [30], 2021^a^	Sleep improvement	App		Yes	
	Jeganathan et al [31], 2022	Physical activity promotion	SMS^b^		Yes	
	NeCamp et al [32], 2020	Physical activity promotion, mental health promotion, and sleep improvement	App		No	
	Spruijt-Metz et al [33], 2022	Physical activity promotion	App		Yes	
	Wang et al [34], 2022	Physical activity promotion and sleep improvement	App		Yes	
	Dowling et al [35], 2022	Gambling reduction	App		Yes	
	Rodda et al [36], 2022	Gambling reduction	App		Yes	
**Evaluate strategies to improve engagement**						
	Bell et al [22], 2020	Alcohol use reduction	App		Yes	
	Bidargaddi et al [37], 2018	Mental health promotion	App		Yes	
	Nahum-Shani et al [38], 2021	Smoking cessation	App		Yes	
	Nordby et al [39], 2022	Mental health promotion	SMS		Yes	
**Evaluate feasibility and acceptability of intervention**						
	Militello et al [40], 2022	Mental health promotion	App		Yes	
	Yang et al [41], 2022	Smoking cessation	App		Yes	
**Describing engagement**						
	Hoel et al [42], 2022	Mental health promotion	App		Yes	
	Valle et al [43], 2020	Dietary lapse prevention or weight management behavior promotion	App		Yes	

^a^This study was also designed to evaluate the feasibility and acceptability of its mobile health intervention.

^b^SMS text messages were delivered as smartphone and smartwatch notifications.

### Synthesis of Results

#### Operationalization of Engagement

##### Overview

Of the 22 included sources, 20 (91%) explicitly included at least 1 measure of engagement; 2 (9%) studies did not claim to measure engagement at all [25,32]; NeCamp et al [32] did not do so because of technical limitations. Though we did not chart the different terms used to refer to participant engagement, we noticed during our full-text screening that some studies did indeed use alternative terms in place of the term “engagement,” such as adherence [27] and investment [30].

##### Measures of Engagement

Table 2 summarizes the measures of engagement used in each study. Across all included studies, system usage data were by far the most frequently used measure of engagement. Sixteen (80%) out of the 20 studies that explicitly measured engagement included at least 1 measure of this category. Generally, researchers used 2 types of system usage data: (1) responsiveness to self-reports, logs, or EMAs [23,24,26,27,29,30,33,35-37,41,42] and (2) access or use of interventions [22,26,33,35,36,39-41,43].

**Table 2:** Measures of engagement used in microrandomized trials of mobile health (mHealth) interventions.

Source		SR^a^ or EMA^b^	SU^c^	Sensor data	Qualitative methods	SM^d^	PP^e^
**Evaluate effect of intervention components**							
	Aguilera et al [23], 2021		✓				
	Battalio et al [24], 2021		✓	✓			
	Goldstein et al [26], 2021		✓				
	Klasnja et al [27], 2021		✓	✓			
	Klasnja et al [28], 2019			✓			
	Kramer et al [29], 2020		✓				
	Latham [30], 2021^f^	✓	✓				
	Jeganathan et al [31], 2022			✓			
	Spruijt-Metz et al [33], 2022		✓	✓			
	Wang et al [34], 2022			✓			
	Dowling et al [35], 2022		✓				
	Rodda et al [36], 2022		✓				
**Evaluate strategies to improve engagement**							
	Bell et al [22], 2020		✓				
	Bidargaddi et al [37], 2018		✓				
	Nahum-Shani et al [38], 2021	✓					
	Nordby et al [39], 2022	✓	✓				
**Evaluate feasibility and acceptability of intervention**							
	Militello et al [40], 2022	✓	✓				
	Yang et al [41], 2022		✓	✓			
**Describing engagement**							
	Hoel et al [42], 2022		✓		✓		
	Valle et al [43], 2020		✓				

^a^SR: self-report data.

^b^EMA: ecological momentary assessment.

^c^SU: system usage data.

^d^SM: social media data.

^e^PP: psychophysiological data.

^f^This study was also designed to evaluate the feasibility and acceptability of its mHealth intervention.

Sensor data were the second most common measure of engagement. Overall, 35% (7/20) of the studies that explicitly measured engagement included at least 1 measure of this category [24,27,28,31,33,34,41]. Wang et al [34], for example, measured the proportion of days in a week that participants wore the study’s FitBit smartwatch to track their step counts and sleep duration.

Engagement was measured via self-reports or EMAs in 20% (4/20) of the studies that explicitly measured engagement [30,38-40]. Latham [30] evaluated a sleep intervention designed to improve the regularity of wake times in college students via prompts. One measure of engagement in this study was participants’ self-reported adherence to the sleep-related suggestions included in the prompt. Nahum-Shani et al [38] proposed to study how prompts to engage in self-regulatory strategies increased engagement in self-regulatory activities; researchers planned to measure engagement as self-reported engagement in self-regulatory activities during the hour after receiving a prompt. In their evaluation of a web-based intervention delivered via SMS text messaging, Nordby et al [39] measured engagement as the self-reported frequency of practicing the coping strategies taught in the web-based intervention. Militello et al [40] assessed the feasibility and acceptability of intervention prompts to encourage engagement in mindfulness activities guided by a mindfulness mobile app. Here, engagement was measured as self-reported performance of a mindfulness activity or exercise in the 24 hours after receiving an intervention prompt.

Only 1 study measured engagement with qualitative methods. In this study, researchers sought to describe engagement with an Acceptance and Commitment Therapy (ACT)–based mobile app in a clinical and a nonclinical sample [42]. The researchers inferred participant engagement by assessing whether participant responses reflected an understanding of the ACT intervention content. The following 3 indicators were used: the identification of the function of behavior, process alignment (whether the content of a given participant’s response is congruent with the core ACT process underlying the intervention prompt received), and the qualitative content of responses.

Only 8 (40%) out of the 20 studies that explicitly measured engagement used >1 method to measure engagement. Interestingly, no study used >2 methods. No studies measured engagement with social media data or psychophysiological measures.

##### Facets of Engagement

Table 3 summarizes the facets of engagement measured by each included study. The physical facet of engagement was the most frequently measured facet of engagement; all 20 studies that explicitly measured engagement included at least 1 measure of this facet [22-24,26-31,33-43]. Multimedia Appendix 2 [22-24,26-31,33-43] provides examples of how this facet of engagement was measured in each included study.

Only 1 study included a measure of the affective facet of engagement [30]. Recall that the affective facet of engagement “captures a wide range of positive affective reactions to a task or activity,” including the “the affective states that may underlie more enduring experiences of attachment, identification, and commitment” [11]. By asking participants how likely they were to complete the intervention (ie, their commitment to the intervention), it could be argued that Latham [30] measured this facet of engagement.

Similarly, only 1 study assessed the cognitive facet of engagement—recall that this involves the “selective attention and processing of information related to a task or activity” [11]. This processing of information related to a task was comprehensively measured by Hoel et al [42] using the qualitative measures described in *Measures of Engagement* section.

**Table 3:** Facets of engagement measured in microrandomized trials of mobile health (mHealth) interventions.

Source		Physical	Affective	Cognitive
**Evaluate effect of intervention components**				
	Aguilera et al [23], 2021	✓		
	Battalio et al [24], 2021	✓		
	Goldstein et al [26], 2021	✓		
	Klasnja et al [27], 2021	✓		
	Klasnja et al [28], 2019	✓		
	Kramer et al [29], 2020	✓		
	Latham [30], 2021^a^	✓	✓	
	Jeganathan et al [31], 2022	✓		
	Spruijt-Metz et al [33], 2022	✓		
	Wang et al [34], 2022	✓		
	Dowling et al [35], 2022	✓		
	Rodda et al [36], 2022	✓		
**Evaluate strategies to improve engagement**				
	Bell et al [22], 2020	✓		
	Bidargaddi et al [37], 2018	✓		
	Nahum-Shani et al [38], 2021	✓		
	Nordby et al [39], 2022	✓		
**Evaluate feasibility and acceptability of intervention**				
	Militello et al [40], 2022	✓		
	Yang et al [41], 2022	✓		
**Describing engagement**				
	Hoel et al [42], 2022	✓		✓
	Valle et al [43], 2020	✓		

^a^This study was also designed to evaluate the feasibility and acceptability of its mHealth intervention.

##### Levels of Engagement

Table 4 summarizes the levels of engagement measured in each included study. Of the 20 studies that explicitly measured engagement, 14 (70%) studies measured Little e only, 2 (10%) studies measured Big E only, and 4 (20%) studies measured both Little e and Big E. Clearly, measures of engagement in MRTs of mHealth interventions are most often Little e measures.

**Table 4:** Levels of engagement measured in microrandomized trials of mobile health (mHealth) interventions.

Source			Little e					Big E		
			Yes or no		Example		Yes or no			Example
**Evaluate effect of intervention components**										
	Aguilera et al [23], 2021	Yes		Response rates to daily mood rating SMS		No			N/A^a^	
	Battalio et al [24], 2021	Yes		If end-of-day logs for smoking are completed		No			N/A	
	Goldstein et al [26], 2021	Yes		Percentage of interventions accessed		No			N/A	
	Klasnja et al [27], 2021	Yes		Adherence to wearing the FitBit		No			N/A	
	Klasnja et al [28], 2019	Yes		Adherence to activity tracker		No			N/A	
	Kramer et al [29], 2020	Yes		Whether participants responded to first message of the chatbot in an intervention conversation		No			N/A	
	Latham [30], 2021^b^	Yes		Percentage of sleep diaries completed		Yes			Self-reported adherence to intervention prompt’s suggestion	
	Jeganathan et al [31], 2022	Yes		Nonadherence with recommendations for watch wear time		No			N/A	
	Spruijt-Metz et al [33], 2022	Yes		Time since FitBit was last worn		No			N/A	
	Wang et al [34], 2022	Yes		Proportion of days that daily step/sleep minutes were provided within a week		No			N/A	
	Dowling et al [35], 2022	Yes		EMA^c^ compliance		No			N/A	
	Rodda et al [36], 2022	Yes		EMA compliance		No			N/A	
**Evaluate strategies to improve engagement**										
	Bell et al [22], 2020	Yes		Whether participants opened the intervention app in the hour after microrandomization		No			N/A	
	Bidargaddi et al [37], 2018	No		N/A		Yes			Whether participants performed the self-monitoring intervention activity	
	Nahum-Shani et al [38], 2021	No		N/A		Yes			Whether participants engaged in self-regulatory activities 1 h after randomization	
	Nordby et al [39], 2022	Yes		Minutes spent on the intervention		Yes			Self-reported frequency of practicing coping strategies taught	
**Evaluate feasibility and acceptability of intervention**										
	Militello et al [40], 2022	Yes		Opening the application		Yes			Self-reported engagement with mindfulness exercises 24 hours after randomization	
	Yang et al [41], 2022	Yes		Percentage of EMAs completed		Yes			Percentage of prompted strategies completed	
**Describing engagement**										
	Hoel et al [42], 2022	Yes		Proportion of submitted and nonblank logs		No			N/A	
	Valle et al [43], 2020	Yes		Proportion of intervention messages viewed before end of day		No			N/A	

^a^N/A: not applicable.

^b^This study was also designed to evaluate the feasibility and acceptability of its mHealth intervention.

^c^EMA: ecological momentary assessment.

#### Determinants of Engagement

Table 5 presents the determinants, moderators, and covariates of engagement studied (if any) in MRTs that assessed or planned to assess engagement. Of the 20 included studies that measured engagement explicitly, 6 (30%) investigated the determinants of participant engagement. Of the 6 studies, 4 (67%) studies were designed to evaluate strategies to improve engagement and investigated the influence of notification-related variables on participant engagement as variables of interest [22,37-39]. The remaining 2 (33%) of the 6 studies were designed to evaluate the effect of intervention components on health outcomes or to describe engagement. The former study assessed a time-based variable as its variable of interest—the causal effect of being in an intervention week on participant engagement [34]. The latter study assessed task-related variables (lapses in self-monitoring and behavioral goal attainment) and an anthropometric variable (weight change) as determinants of participant engagement [43].

Of the 6 studies, only 3 (50%) studies designed to evaluate strategies to improve engagement investigated how the determinants of engagement were moderated. Two of these studies exclusively examined the moderating effect of time-related variables [22,37]. Concretely, Bell et al [22] investigated how the causal effect of sending a push notification (vs not sending it) on engagement was moderated by the number of days in the study. Bidargaddi et al [37], by contrast, investigated if the causal effect of sending (vs not sending) a push notification on engagement was moderated by the number of weeks in the study or by the day of the week (sent on a weekday or a weekend). The third study of this trio planned to study the moderating effect of a comprehensive set of physiological and psychosocial moderators representing vulnerability and receptivity, in addition to time-related moderators [38].

**Table 5:** Determinants, moderators, and covariates of engagement assessed in microrandomized trials of mobile health (mHealth) interventions.

Source		Determinants	Moderators	Covariates
**Evaluate effect of intervention components**				
	Wang et al [34], 2022	Time related	N/A^a^	N/A
**Evaluate strategies to improve engagement**				
	Bell et al [22], 2020	Notification related	Time related	Demographic, time related, and health behavior related
	Bidargaddi et al [37], 2018	Notification related	Time related	Time related, notification related, and task related
	Nahum-Shani et al [38], 2021	Notification related	Psychological, societal, health behavior related, contextual, time related, physiological, and demographic	Demographic and time related
	Nordby et al [39], 2022	Notification related	N/A	N/A
**Describing engagement**				
	Valle et al [43], 2020	Task related, anthropometric	N/A	Time related, notification related, and anthropometric

^a^N/A: not applicable.

## Discussion

### Principal Findings

In this scoping review, we aimed to better understand the state of participant engagement measurement in MRTs of mHealth interventions. To do so, we quantified the proportion of existing and planned studies that have explicitly assessed engagement and investigated how engagement has been operationalized in these MRTs. Of the 22 eligible studies indexed by our search, 20 (91%) studies included at least 1 explicit measure of engagement. Overall, our findings suggest that MRTs of mHealth interventions have operationalized engagement in overly narrow terms. We also sought to identify the factors that have been studied as determinants of engagement in MRTs of mHealth interventions. We found that out of the 20 studies that measured engagement explicitly, only 6 (30%) studies investigated the determinants of engagement. Even fewer attempts had been made to investigate the moderators of engagement.

### Operationalization of Engagement

#### Measures of Engagement

Objective measures of engagement—in particular, system usage data (16/20, 80%) and sensor data (7/20, 35%)—were the most common methods of measuring engagement in MRTs of mHealth interventions. The relative popularity of measuring engagement with objective measures, especially system usage data, in MRTs of mHealth interventions is not surprising. System usage has been a central focus in the extant mHealth intervention literature [16]. In fact, it is one of the most common measures of engagement in mHealth interventions [10,44,45]. Subjective measures of engagement, by contrast, were far less common: self-report or EMA (4/20, 20%) and qualitative methods (1/20, 5%). Unfortunately, the lack of attention to the subjective experiences of participants in engagement measurement is not unique to MRTs of mHealth interventions [14,17]. Surprisingly, only 8 (40%) out of the 20 studies measured engagement using >1 method (no study used >2 methods). Of these 8 studies, only half (4/8, 50%) used both subjective and objective measures of engagement.

Taken together, these findings highlight a pressing need for future MRTs of mHealth interventions to diversify the methods of engagement used; the aforementioned lack of diversity does not seem limited to mHealth interventions evaluated using MRTs [14]. Researchers should keep in mind that subjective and objective methods are complementary, not competing, methods to measure engagement—subjective methods provide unique information about participant engagement that objective methods do not capture and vice versa [13,14]. Let us consider the distinction between qualitative and sensor data measures of engagement. Using qualitative methods, we may glean interesting insights about how a participant feels about an intervention or how cognitively invested they are in the intervention. This is certainly not possible for sensor data extracted from a pedometer. However, with said sensor data, it is possible to obtain detailed information (unobtrusively) about health behavior participation and how it fluctuates over time. We recommend that future MRTs of mHealth interventions adopt a multimethod approach to engagement measurement [13] such that engagement data from several subjective and objective measures are collected and interpreted.

#### Facets of Engagement

In this review, we found that the physical facet of engagement was the dominant kind of engagement measured in MRTs of mHealth interventions. Indeed, all 20 studies included at least 1 explicit measure of this facet. Surprisingly, the affective and cognitive facets of engagement were only measured by 1 study each. Clearly, our findings suggest an imbalance in the *kinds* of engagement measured and that researchers’ conceptualizations of engagement, and consequently their operationalizations of engagement, are largely constrained to intervention-related task or activity performance. Given that self-report and qualitative measures of engagement are best suited to measure the affective and cognitive facets of engagement, we cannot rule out that this imbalance is a product of the lack of diversity in methods of measuring engagement described in *Measures of Engagement* subsection in the *Discussion* section.

From the theoretical position that engagement is a multidimensional latent construct composed of physical, affective, and cognitive facets, this imbalance is particularly worrying because it signals that the construct of engagement is not being adequately measured in MRTs of mHealth interventions. Scholars who adopt this position generally agree that no facet of engagement alone can constitute engagement. Instead, they concur that engagement involves the physical, emotional, and cognitive energies of a person working in concert [11]. Therefore, without measuring all 3 facets of engagement, it is not possible to accurately identify how engaged participants are with a task. We hope that this review will draw attention to this gap in engagement measurement and encourage future MRTs of mHealth interventions to incorporate more measures of the affective and cognitive facets of engagement.

On a related note, although an assessment of the quality of engagement measurement in MRTs of mHealth interventions is beyond the scope of this review, we did observe that many included studies relied on single items to measure engagement. Estimates of reliability were also rarely (if ever) reported. As single items have a bad reputation for being unreliable measures of psychological constructs [46], we encourage researchers to clearly report estimates of reliability (such as test-retest reliability) so that readers can evaluate for themselves how much variation in “engagement scores” can be attributed to measurement error.

#### Levels of Engagement

The distinction between Little e and Big E is an important consideration when studying engagement in digital health interventions. Recall that Little e and Big E can be construed as 2 distinct answers to the question “Engagement with what?” [11]. Our findings suggest that most explicit measures of engagement in MRTs of mHealth interventions are Little e measures (measures of engagement with the mHealth intervention) and that only a handful of studies have measured engagement with the health behavior of interest (or Big E).

Although this review focuses on explicit claims of engagement measurement, a careful analysis of the outcome measures used in all 22 studies makes it clear that many of these outcomes qualify as Big E measures, even though they were not explicitly conceptualized as such [15]. This was observed in 12 studies [24-29,31-36]. All 12 studies were designed to evaluate the effects of intervention components. Most of these studies measured the physical aspect of engagement using sensor data. If we account for such studies, we may conclude that all 22 studies of mHealth interventions included in this review included at least one measure of engagement and that out of the 22 MRTs of mHealth interventions included here, 4 (18%) studies measured Little e only, 4 (18%) studies measured Big E only, and 14 (64%) studies measured both Little e and Big E (Multimedia Appendix 3 [22-43]). It was difficult for us to decide whether the outcome measures of these 12 studies should be deemed measures of engagement in this review. Our concern stems from the fact that the inclusion of these outcomes as measures of engagement hinges on our use of the Little e and Big E distinction to understand how engagement has been operationalized. If this distinction was not invoked, there would be no clear evidence from these 12 studies to suggest that these outcome measures are measures of engagement or that the authors themselves considered them to be measures of engagement. Let us consider the engagement-related information extracted from Goldstein et al [26], which is one of the 12 studies. The outcome measure of this study, whether a dietary lapse was experienced since the last EMA, is a clear-cut measure of Big E. However, it was not included in the authors’ own list of engagement measures stated in the paper. If the authors themselves do not conceptualize these outcomes as measures of engagement, would it be appropriate to include these outcomes as measures of engagement in this scoping review? Even if we were to include this outcome as a measure of engagement, can we assume that the underlying motivations of Goldstein et al [26]—in terms of modeling decisions and decisions about the study design—are similar to those of researchers who explicitly frame health behavior outcomes as measures of engagement? This is important because we cannot rule out the possibility that researchers’ choice of causal effects, moderators, and control variables are at least partly influenced by how they conceptualize outcome measures. On the basis of these considerations, we decided not to consider the outcome measures of these 12 studies as measures of engagement in this scoping review.

Nevertheless, our findings clearly suggest the need for future MRTs of mHealth interventions to strike a balance between Little e and Big E measurement or at least be more intentional and explicit with Big E measurement (especially when using sensor data as an outcome measure). As the field begins to recognize that sustained engagement is not always required for participants to experience the intended health outcomes of an intervention [13], we encourage researchers to find this balance so that they can gain a sense of effective engagement in the interventions they develop—the sufficient amount of engagement needed to attain the intended outcome of the intervention [11,14].

### Determinants of Engagement

We found that very few studies investigated the determinants of engagement (6/20, 30% of the studies that measured engagement). In studies that did assess the determinants of engagement, notification-related causal effects were most common. This is likely attributable to the fact that most of these studies were designed to evaluate strategies to improve engagement [22,37-39]. Even fewer studies (3/6, 50%) assessed the moderators of engagement. Although all 3 studies assessed time-related (time-variant) moderators such as the number of days in the study or the day of the week, only 1 study [38] planned to investigate time-invariant moderators (such as psychological or social variables) in addition to the time-related moderators. These findings suggest that there is a striking lack of attention to how engagement is determined and to the effect of time-invariant psychosocial moderators on engagement in existing MRTs of mHealth interventions. To advance our understanding of engagement in the multicomponent and adaptive mHealth interventions tested by MRTs, it is necessary for future MRTs to address this research gap.

To begin addressing this research gap, we recommend that researchers adopt existing theoretical frameworks to guide their selection of the determinants and moderators of participant engagement in MRTs of mHealth interventions. If widely adopted, this approach should ensure some semblance of parity in the kinds of determinants and moderators of engagement studied across MRTs and provide researchers with a common taxonomy (or at least a common language) to guide their inquiry. With this, researchers can compare and synthesize results from different MRTs to better understand how engagement is modulated across mHealth interventions tested with MRTs.

Researchers can consider studying the determinants and moderators of engagement through the lens of participant engagement frameworks. Recently, Nahum-Shani et al [11] proposed the affect-integration-motivation and attention-​context-translation framework for participant engagement. In this paper, they outlined 3 areas, namely, attention, contextual influences, and the translation of motivation to behavior (attention-context-translation), that might influence the neural-based process (affect-integration-motivation) of how engagement with a task (eg, walking) is realized through engagement with a stimulus (eg, a prompt to take a walk). It would be interesting for future MRTs to examine how constructs from each of these 3 areas contribute to participant engagement. Alternatively, researchers can consider selecting theoretically relevant determinants and moderators from the Big Five Personality trait framework [47], which is composed of trait openness, conscientiousness, extraversion, agreeableness, and neuroticism. This approach might be a good first step toward clarifying the role of individual differences in participant engagement, considering the lack of attention given to the psychological characteristics of participants in the extant MRT literature and the relevance of personality to health behaviors and outcomes [48]. Researchers should pay particular attention to the role of conscientiousness as it seems to be the most relevant to mHealth engagement [49] and it has been consistently linked to positive health behaviors [50,51]. These 2 frameworks are by no means exhaustive. We encourage researchers interested in understanding the determinants and moderators of engagement to seek out other appropriate frameworks to advance this line of research.

### Limitations

There are 3 notable limitations of this scoping review. First, at the time of conducting our database searches, there was no available Medical Subject Heading in PubMed for MRTs (or equivalent controlled vocabularies for other databases). Therefore, our database searches might not have picked up papers and protocols that did not use the phrase “micro-randomised trial” or “micro-randomized trial” as a keyword or in their title and abstract. Nevertheless, we believe that the main findings of this scoping review still hold true, as our database and manual searches would have indexed most mHealth intervention MRTs planned and conducted to date. Second, we did not use existing frameworks such as the Frequency, Intensity, Time, and Type principle [14] to further categorize engagement measured using system usage data. This has been done in previous scoping reviews [17] and is necessary to obtain a nuanced understanding of engagement measurement in mHealth interventions. Unfortunately, we were not able to do so, as some studies and protocols did not clearly operationalize their measurement of engagement in exact terms. Finally, it must be noted that because of the inclusion and exclusion criteria, we were not able to include several well-designed MRTs in this review because they were not strictly evaluations of mHealth interventions—they were designed either to evaluate digital but not mHealth interventions [52,53] or to evaluate engagement strategies only [54-56]. To fully understand the extent of engagement measurement in digital health interventions evaluated by MRTs, we encourage future reviews to broaden their inclusion and exclusion criteria to include these 2 types of evidence sources.

### Conclusions

In this scoping review, we demonstrate that although most MRTs of mHealth interventions have measured engagement explicitly, they have operationalized engagement in overly narrow terms; there is an overemphasis on using objective measurements of engagement, measuring the physical facet of engagement, and measuring engagement with the mHealth intervention (as opposed to engagement with the health behavior of interest). There is also a lack of attention to how engagement is determined and moderated in these existing trials. We hope that by mapping the state of engagement measurement, this review will encourage researchers to pay more attention to these issues when planning engagement measurement in future MRTs. Although these issues are by no means unique to mHealth interventions evaluated with MRTs, the relative infancy of the MRT design suggests that there is still time and opportunity for the field to course correct and establish best practices for the measurement of engagement in MRTs of mHealth interventions.

## Appendix

Multimedia Appendix 1 All charted data for the included evidence sources.

Multimedia Appendix 2 Engagement measures of the included evidence sources organized according to the method of engagement measurement used, the facets of engagement measured, and the levels of engagement measured.

Multimedia Appendix 3 Levels of engagement measured in microrandomized trials of mobile health (mHealth) interventions if 12 outcome measures are considered measures of Big E.

## Abbreviations

ACT: Acceptance and Commitment Therapy
EMA: ecological momentary assessment
mHealth: mobile health
MRT: microrandomized trial
OSF: Open Science Framework
PRISMA-ScR: Preferred Reporting Items for Systematic Reviews and Meta-Analysis extension for Scoping Reviews
RCT: randomized controlled trial
