# Supplementary material for: Participant Engagement in Microrandomized Trials of mHealth Interventions: Scoping Review
Source: JMIR Mhealth Uhealth. 2023 May 22;11:e44685. doi: 10.2196/44685 (PMC10242468; doi:10.2196/44685)
Supplement: Multimedia Appendix 3 [file mhealth_v11i1e44685_app3.pdf]

This is a Multimedia Appendix to a full manuscript published in the JMIR mHealth uHealth.
For full copyright and citation information see http://dx.doi.org/10.2196/jmir.44685

Multimedia Appendix. Levels of engagement measured in micro-randomized trials of mHealth interventions.[a-b]

	Source	Little e		Big E	
		Y/N	Example	Y/N	Example
**Evaluate effect of intervention component(s)**					
	Aguilera et al. (2021)	Y	Response rates to daily mood rating SMS	N	
	Battalio et al. (2021)	Y	If end-of-day logs for smoking are completed	Y[b]	Probability of smoking episode
	Figueroa et al. (2022)	N		Y[b]	Daily change in the number of steps taken
	Goldstein et al. (2021)	Y	% of interventions accessed	Y[b]	Whether participant experience a dietary lapse
	Klasnja et al. (2021)	Y	Adherence to wearing the FitBit	Y[b]	Step count in the 30 minutes after randomization
	Klasnja et al. (2019)	Y	Adherence to activity tracker	Y[b]	Step count in the 30 minutes after randomization
	Kramer et al. (2020)	Y	Whether participants responded to first message of the chatbot in an intervention conversation	Y[b]	Proportion of days that daily step goal achieved
	Latham (2021)[a]	Y	% of sleep diaries completed	Y	Self-reported adherence to intervention

					prompt's suggestion
	Jeganathan et al. (2022)	Y	Nonadherence with recommendations for watch wear time	Y[b]	Average step count within one hour after notification
	NeCamp et al. (2020)	N		Y[b]	Average daily step count
	Spruijt-Metz et al. (2022)	Y	Time since FitBit last worn	Y[b]	Daily step count
	Wang et al. (2022)	Y	Proportion of days that daily step/sleep minutes were provided within a week	Y[b]	Weekly average daily step count
	Dowling et al. (2022)	Y	EMA compliance	Y[b]	Probability of subsequent gambling episode
	Rodda et al. (2022)	Y	EMA compliance	Y[b]	Adherence to gambling expenditure limits
**Evaluate strategies to improve engagement**					
	Bell et al. (2020)	Y	Whether participants opened the intervention app in the hour after micro-randomization	N	
	Bidargaddi et al. (2018)	N		Y	Whether participants performed the self-monitoring intervention activity
	Nahum-Shani et al. (2021)	N		Y	Whether participants engaged in self-regulatory

					activities one hour after randomization
Evaluate feasibility and acceptability of intervention	Nordby et al. (2022)	Y	Minutes spent online in the intervention	Y	Self-reported frequency of practicing coping strategies taught
	Militello et al. (2022)	Y	Opening the application	Y	Self-reported engagement with mindfulness exercises 24 hours after randomization
Describing engagement	Yang et al. (2022)	Y	% of EMAs completed	Y	% of prompted strategies completed
	Hoel et al. (2022)	Y	Proportion of submitted/non-blank logs	N	
	Valle et al. (2020)	Y	Proportion of intervention messages viewed before end of day	N	

[a]This study was also designed to evaluate the feasibility and acceptability of its mHealth intervention.
[b]The authors did not explicitly claim to measure engagement with health behavior. However, through the lens of the Little e/Big E distinction, an outcome measure of this study can be construed to index Big E or engagement with the health behavior of interest.
